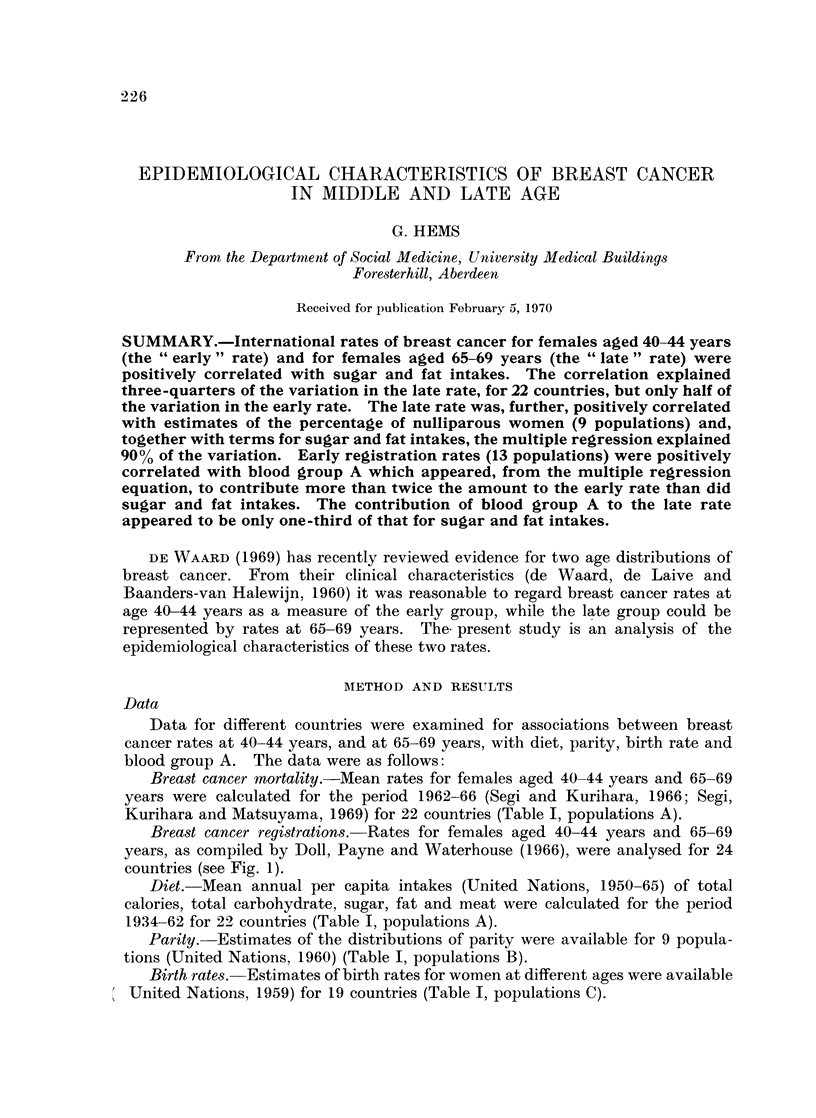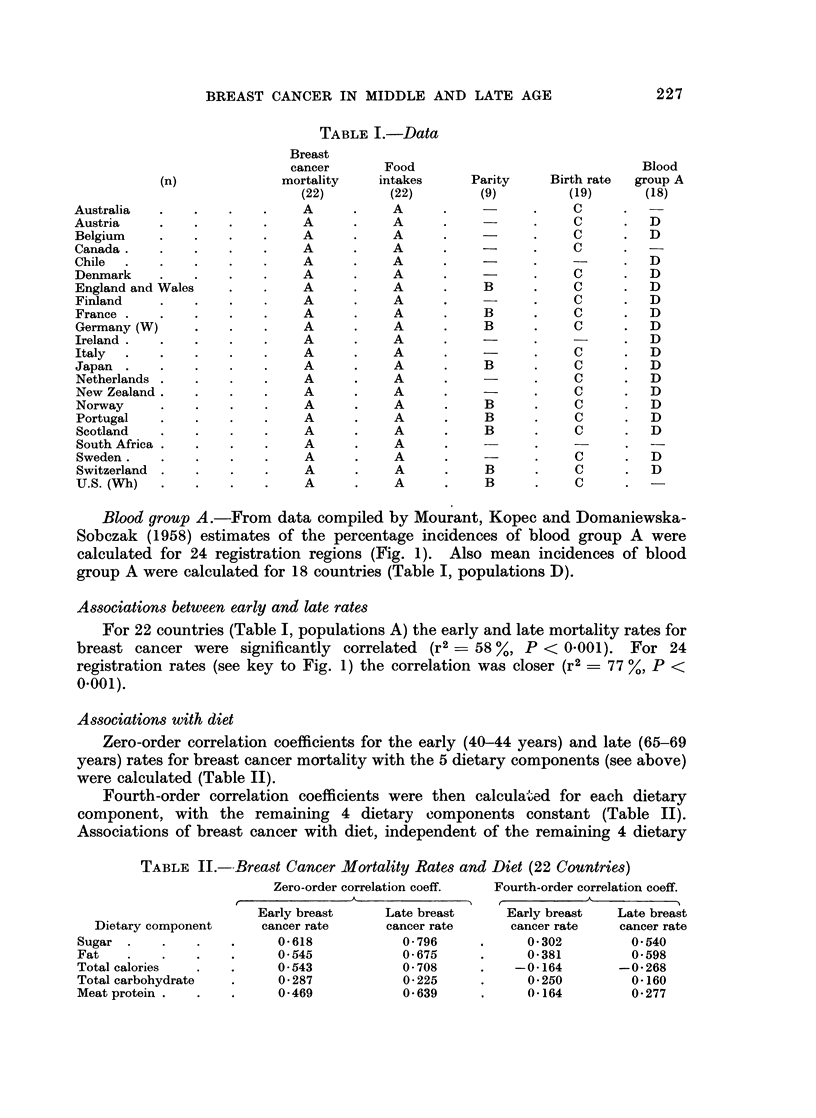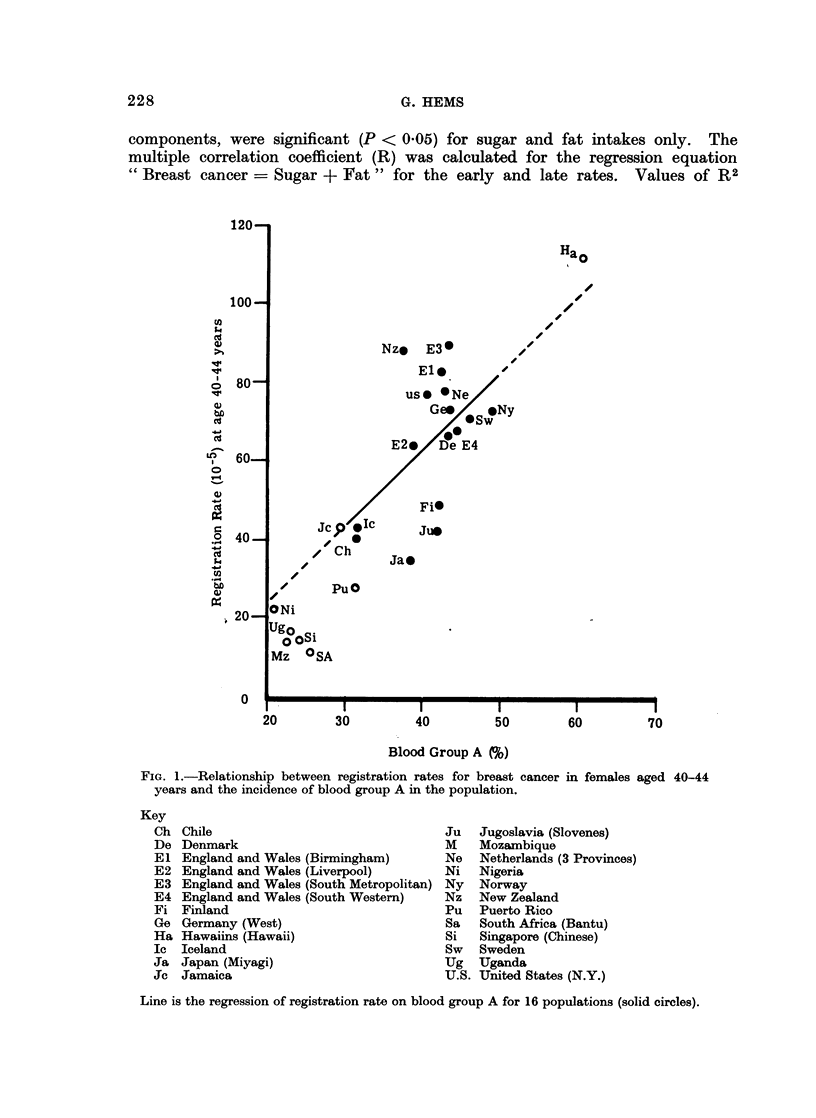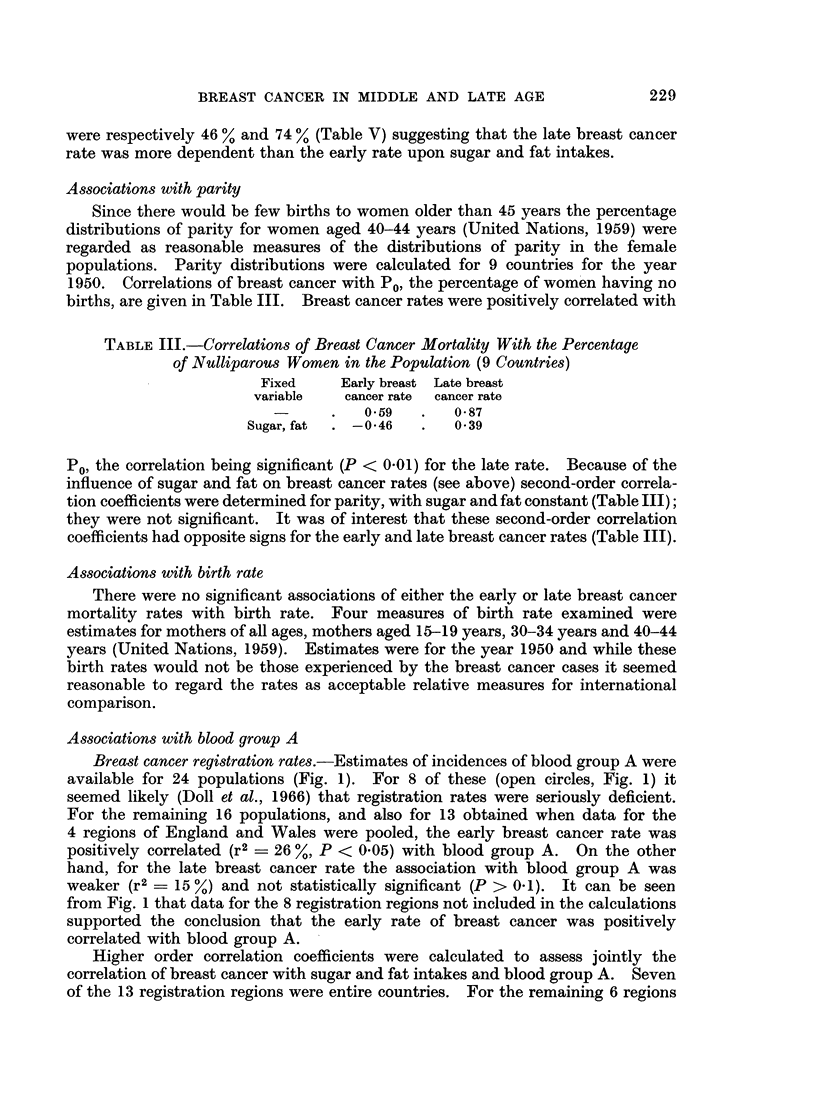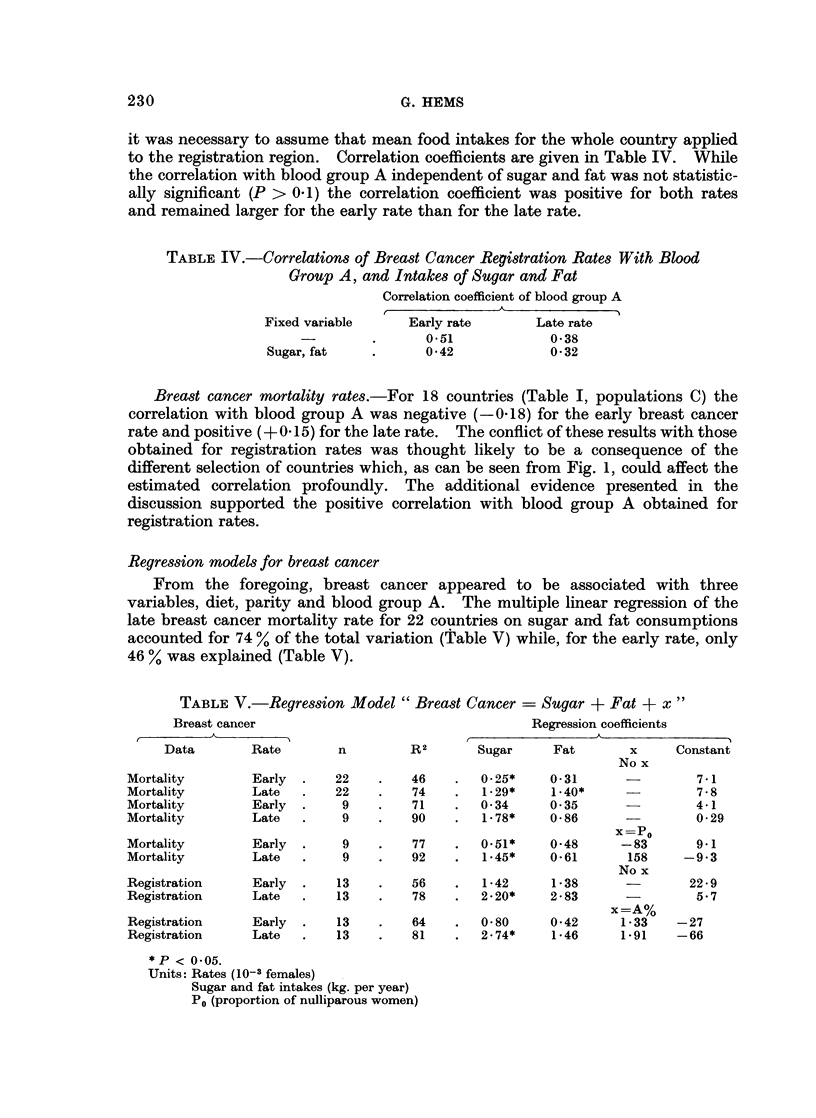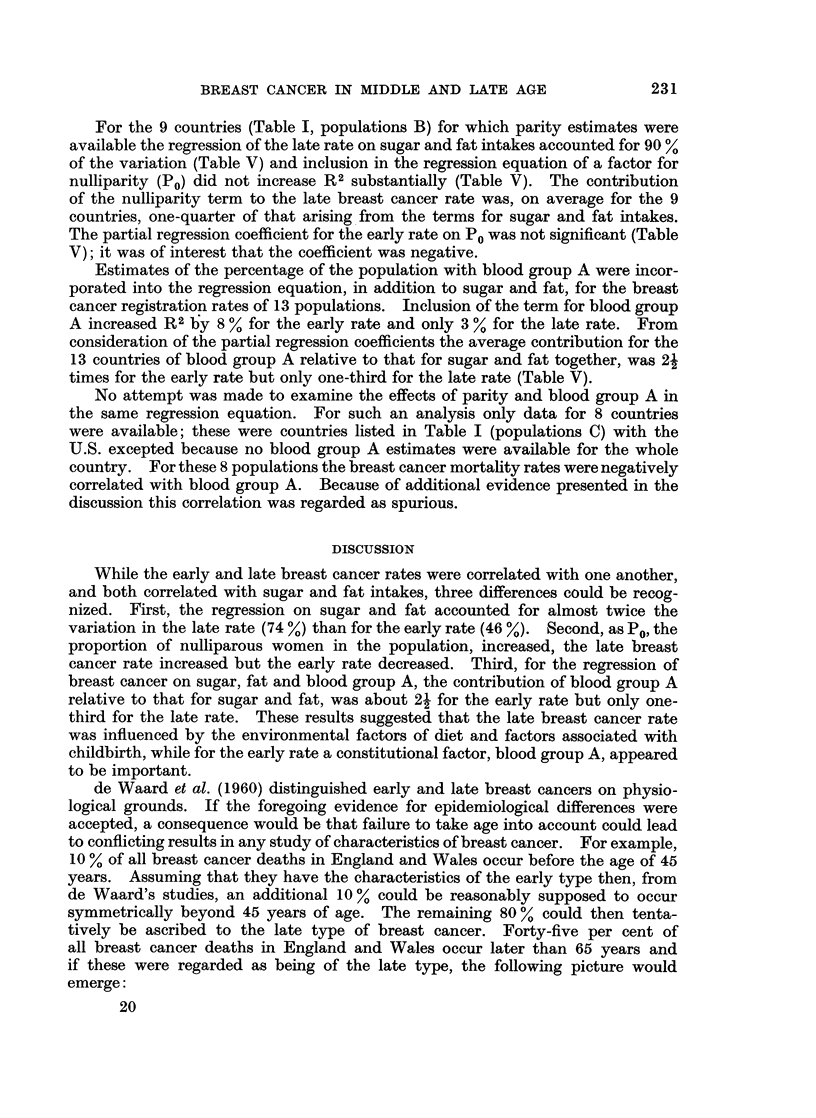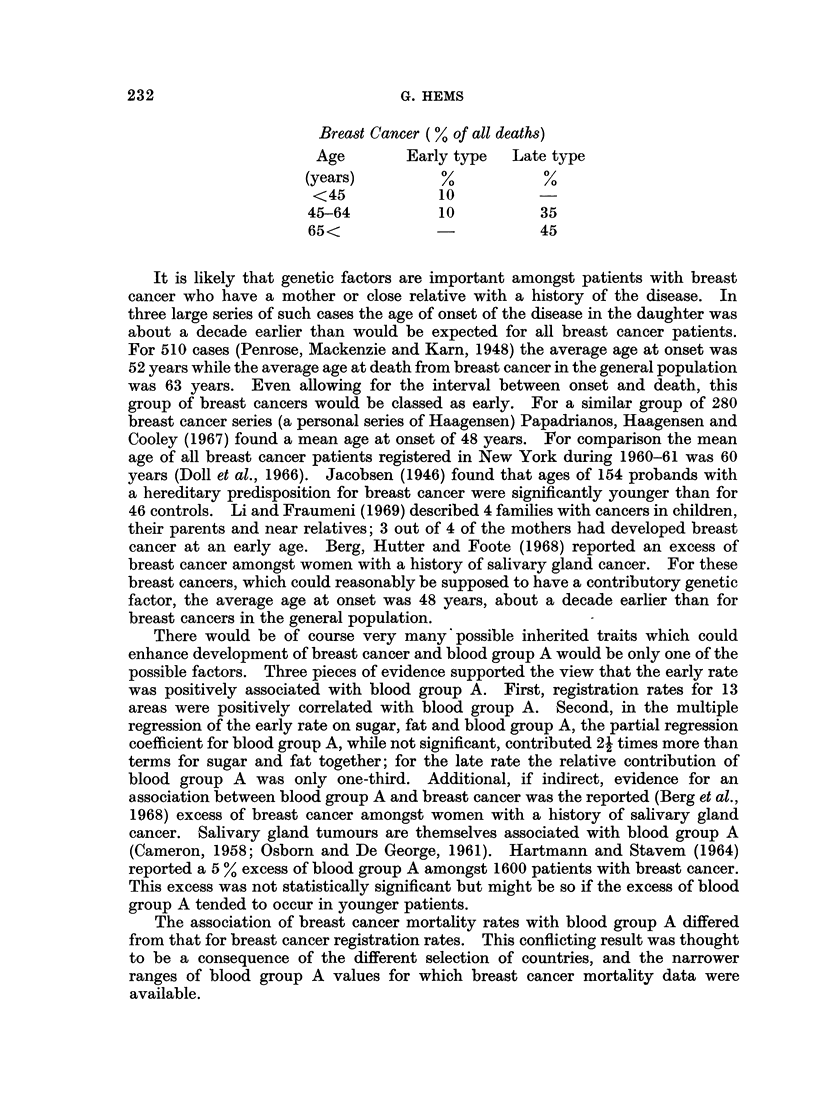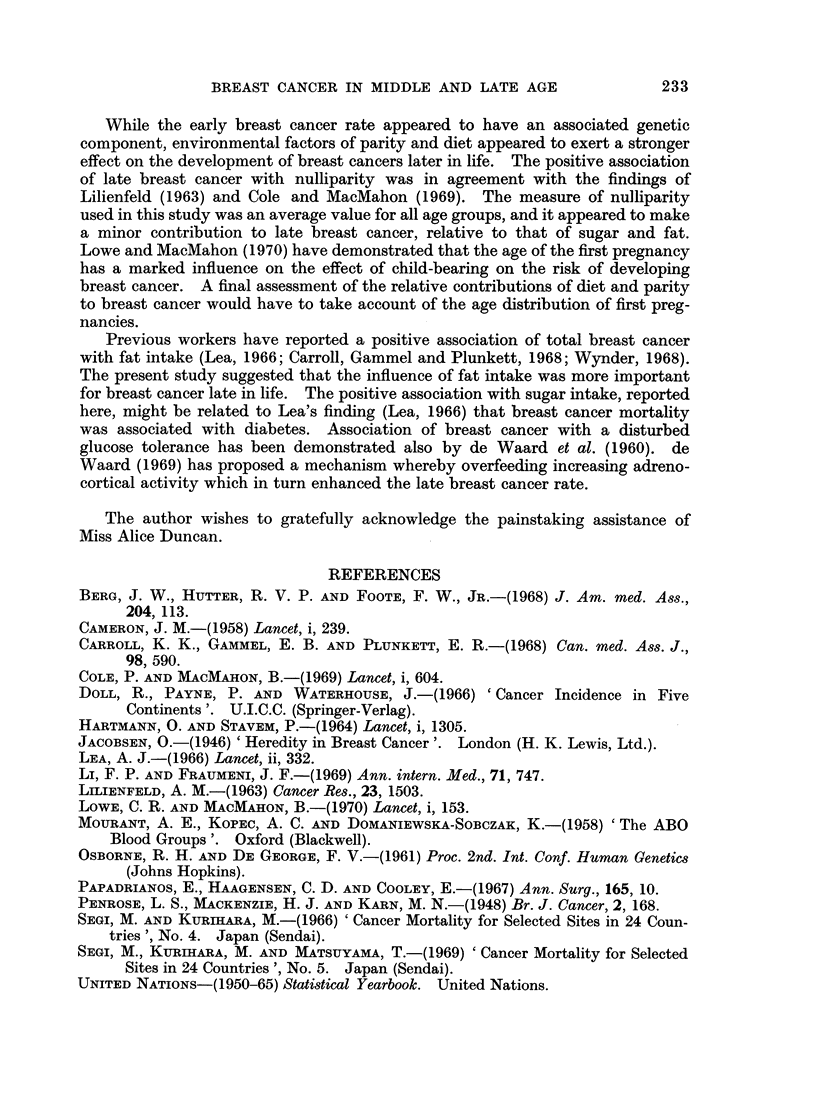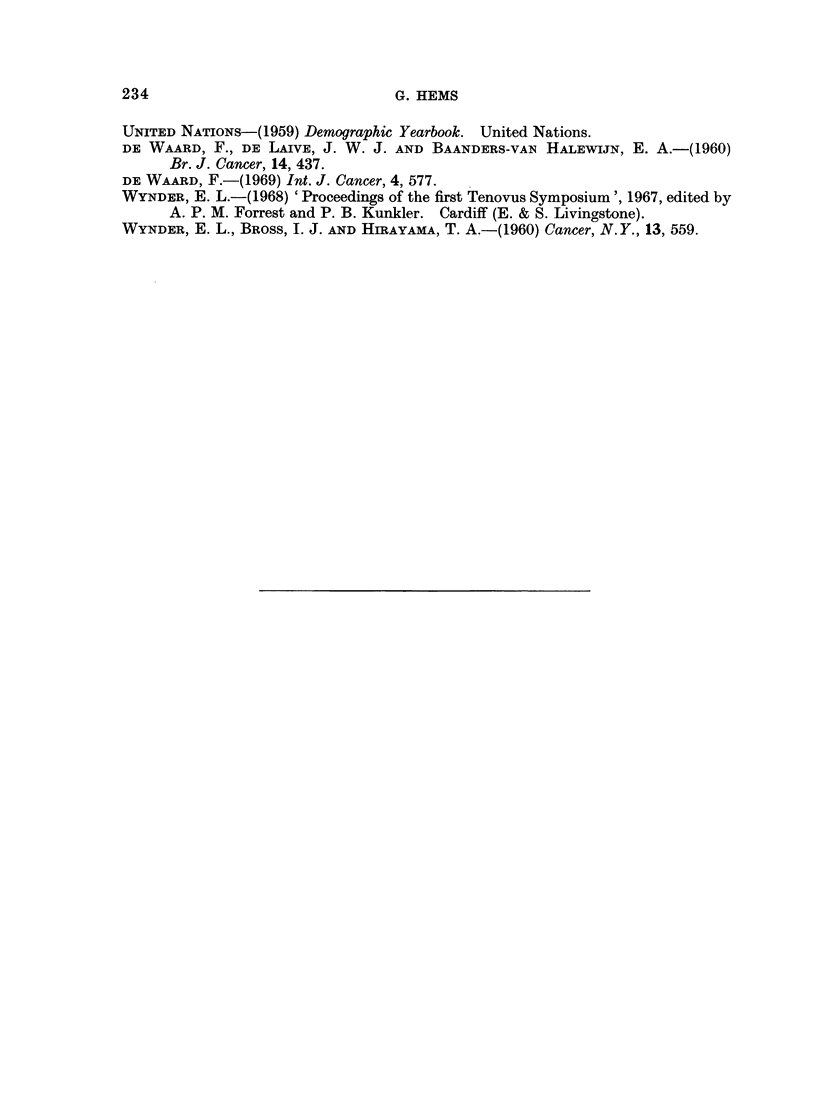# Epidemiological Characteristics of Breast Cancer in Middle and Late Age

**DOI:** 10.1038/bjc.1970.26

**Published:** 1970-06

**Authors:** G. Hems

## Abstract

International rates of breast cancer for females aged 40-44 years (the “early” rate) and for females aged 65-69 years (the “late” rate) were positively correlated with sugar and fat intakes. The correlation explained three-quarters of the variation in the late rate, for 22 countries, but only half of the variation in the early rate. The late rate was, further, positively correlated with estimates of the percentage of nulliparous women (9 populations) and, together with terms for sugar and fat intakes, the multiple regression explained 90% of the variation. Early registration rates (13 populations) were positively correlated with blood group A which appeared, from the multiple regression equation, to contribute more than twice the amount to the early rate than did sugar and fat intakes. The contribution of blood group A to the late rate appeared to be only one-third of that for sugar and fat intakes.


### EPIDEMIOLOGICAL CHARACTERISTICS OF BREAST CANCER IN MIDDLE AND LATE AGE

G. HEMS

*From the Department of Social Medicine, University Medical Buildings Foresterhill, Aberdeen*

Received for publication February 5, 1970

**SUMMARY.—International rates of breast cancer for females aged 40–44 years (the " early " rate) and for females aged 65–69 years (the " late " rate) were positively correlated with sugar and fat intakes. The correlation explained three-quarters of the variation in the late rate, for 22 countries, but only half of the variation in the early rate. The late rate was, further, positively correlated with estimates of the percentage of nulliparous women (9 populations) and, together with terms for sugar and fat intakes, the multiple regression explained 90% of the variation. Early registration rates (13 populations) were positively correlated with blood group A which appeared, from the multiple regression equation, to contribute more than twice the amount to the early rate than did sugar and fat intakes. The contribution of blood group A to the late rate appeared to be only one-third of that for sugar and fat intakes.**

DE WAARD (1969) has recently reviewed evidence for two age distributions of breast cancer. From their clinical characteristics (de Waard, de Laive and Baanders-van Halewijn, 1960) it was reasonable to regard breast cancer rates at age 40–44 years as a measure of the early group, while the late group could be represented by rates at 65–69 years. The present study is an analysis of the epidemiological characteristics of these two rates.

### METHOD AND RESULTS

### *Data*

Data for different countries were examined for associations between breast cancer rates at 40–44 years, and at 65–69 years, with diet, parity, birth rate and blood group A. The data were as follows:

*Breast cancer mortality.*—Mean rates for females aged 40–44 years and 65–69 years were calculated for the period 1962–66 (Segi and Kurihara, 1966; Segi, Kurihara and Matsuyama, 1969) for 22 countries (Table I, populations A).

*Breast cancer registrations.*—Rates for females aged 40–44 years and 65–69 years, as compiled by Doll, Payne and Waterhouse (1966), were analysed for 24 countries (see Fig. 1).

*Diet.*—Mean annual per capita intakes (United Nations, 1950–65) of total calories, total carbohydrate, sugar, fat and meat were calculated for the period 1934–62 for 22 countries (Table I, populations A).

*Parity.*—Estimates of the distributions of parity were available for 9 populations (United Nations, 1960) (Table I, populations B).

*Birth rates.*—Estimates of birth rates for women at different ages were available (United Nations, 1959) for 19 countries (Table I, populations C).

TABLE I.—*Data*

| (n) | Breast cancer mortality (22) | Food intakes (22) | Parity (9) | Birth rate (19) | Blood group A (18) |
|---|---|---|---|---|---|
| Australia | A | A | — | C | — |
| Austria | A | A | — | C | D |
| Belgium | A | A | — | C | D |
| Canada | A | A | — | C | — |
| Chile | A | A | — | — | D |
| Denmark | A | A | — | C | D |
| England and Wales | A | A | B | C | D |
| Finland | A | A | — | C | D |
| France | A | A | B | C | D |
| Germany (W) | A | A | B | C | D |
| Ireland | A | A | — | — | D |
| Italy | A | A | — | C | D |
| Japan | A | A | B | C | D |
| Netherlands | A | A | — | C | D |
| New Zealand | A | A | — | C | D |
| Norway | A | A | B | C | D |
| Portugal | A | A | B | C | D |
| Scotland | A | A | B | C | D |
| South Africa | A | A | — | — | — |
| Sweden | A | A | — | C | D |
| Switzerland | A | A | B | C | D |
| U.S. (Wh) | A | A | B | C | — |

*Blood group A*.—From data compiled by Mourant, Kopec and Domaniewska-Sobczak (1958) estimates of the percentage incidences of blood group A were calculated for 24 registration regions (Fig. 1). Also mean incidences of blood group A were calculated for 18 countries (Table I, populations D).

*Associations between early and late rates*

For 22 countries (Table I, populations A) the early and late mortality rates for breast cancer were significantly correlated ($r^2 = 58\%$, $P < 0{\cdot}001$). For 24 registration rates (see key to Fig. 1) the correlation was closer ($r^2 = 77\%$, $P < 0{\cdot}001$).

*Associations with diet*

Zero-order correlation coefficients for the early (40–44 years) and late (65–69 years) rates for breast cancer mortality with the 5 dietary components (see above) were calculated (Table II).

Fourth-order correlation coefficients were then calculated for each dietary component, with the remaining 4 dietary components constant (Table II). Associations of breast cancer with diet, independent of the remaining 4 dietary

TABLE II.—*Breast Cancer Mortality Rates and Diet* (*22 Countries*)

| | Zero-order correlation coeff. | | Fourth-order correlation coeff. | |
|---|---|---|---|---|
| Dietary component | Early breast cancer rate | Late breast cancer rate | Early breast cancer rate | Late breast cancer rate |
| Sugar | 0·618 | 0·796 | 0·302 | 0·540 |
| Fat | 0·545 | 0·675 | 0·381 | 0·598 |
| Total calories | 0·543 | 0·708 | −0·164 | −0·268 |
| Total carbohydrate | 0·287 | 0·225 | 0·250 | 0·160 |
| Meat protein | 0·469 | 0·639 | 0·164 | 0·277 |

components, were significant ($P < 0{\cdot}05$) for sugar and fat intakes only. The multiple correlation coefficient (R) was calculated for the regression equation " Breast cancer = Sugar + Fat " for the early and late rates. Values of $R^2$

FIG. 1.—Relationship between registration rates for breast cancer in females aged 40–44 years and the incidence of blood group A in the population.

Key

| | | | |
|---|---|---|---|
| Ch | Chile | Ju | Jugoslavia (Slovenes) |
| De | Denmark | M | Mozambique |
| E1 | England and Wales (Birmingham) | Ne | Netherlands (3 Provinces) |
| E2 | England and Wales (Liverpool) | Ni | Nigeria |
| E3 | England and Wales (South Metropolitan) | Ny | Norway |
| E4 | England and Wales (South Western) | Nz | New Zealand |
| Fi | Finland | Pu | Puerto Rico |
| Ge | Germany (West) | Sa | South Africa (Bantu) |
| Ha | Hawaiins (Hawaii) | Si | Singapore (Chinese) |
| Ic | Iceland | Sw | Sweden |
| Ja | Japan (Miyagi) | Ug | Uganda |
| Jc | Jamaica | U.S. | United States (N.Y.) |

Line is the regression of registration rate on blood group A for 16 populations (solid circles).

were respectively 46 % and 74 % (Table V) suggesting that the late breast cancer rate was more dependent than the early rate upon sugar and fat intakes.

*Associations with parity*

Since there would be few births to women older than 45 years the percentage distributions of parity for women aged 40–44 years (United Nations, 1959) were regarded as reasonable measures of the distributions of parity in the female populations. Parity distributions were calculated for 9 countries for the year 1950. Correlations of breast cancer with $P_0$, the percentage of women having no births, are given in Table III. Breast cancer rates were positively correlated with $P_0$, the correlation being significant ($P < 0{\cdot}01$) for the late rate. Because of the influence of sugar and fat on breast cancer rates (see above) second-order correlation coefficients were determined for parity, with sugar and fat constant (Table III); they were not significant. It was of interest that these second-order correlation coefficients had opposite signs for the early and late breast cancer rates (Table III).

TABLE III.—*Correlations of Breast Cancer Mortality With the Percentage of Nulliparous Women in the Population* (9 *Countries*)

| Fixed variable | Early breast cancer rate | Late breast cancer rate |
|---|---|---|
| — | 0·59 | 0·87 |
| Sugar, fat | −0·46 | 0·39 |

*Associations with birth rate*

There were no significant associations of either the early or late breast cancer mortality rates with birth rate. Four measures of birth rate examined were estimates for mothers of all ages, mothers aged 15–19 years, 30–34 years and 40–44 years (United Nations, 1959). Estimates were for the year 1950 and while these birth rates would not be those experienced by the breast cancer cases it seemed reasonable to regard the rates as acceptable relative measures for international comparison.

*Associations with blood group A*

*Breast cancer registration rates.*—Estimates of incidences of blood group A were available for 24 populations (Fig. 1). For 8 of these (open circles, Fig. 1) it seemed likely (Doll *et al.*, 1966) that registration rates were seriously deficient. For the remaining 16 populations, and also for 13 obtained when data for the 4 regions of England and Wales were pooled, the early breast cancer rate was positively correlated ($r^2 = 26\%$, $P < 0{\cdot}05$) with blood group A. On the other hand, for the late breast cancer rate the association with blood group A was weaker ($r^2 = 15\%$) and not statistically significant ($P > 0{\cdot}1$). It can be seen from Fig. 1 that data for the 8 registration regions not included in the calculations supported the conclusion that the early rate of breast cancer was positively correlated with blood group A.

Higher order correlation coefficients were calculated to assess jointly the correlation of breast cancer with sugar and fat intakes and blood group A. Seven of the 13 registration regions were entire countries. For the remaining 6 regions it was necessary to assume that mean food intakes for the whole country applied to the registration region. Correlation coefficients are given in Table IV. While the correlation with blood group A independent of sugar and fat was not statistically significant ($P > 0\cdot1$) the correlation coefficient was positive for both rates and remained larger for the early rate than for the late rate.

TABLE IV.—*Correlations of Breast Cancer Registration Rates With Blood Group A, and Intakes of Sugar and Fat*

| Fixed variable | Correlation coefficient of blood group A | |
|---|---|---|
| | Early rate | Late rate |
| — . | 0·51 | 0·38 |
| Sugar, fat . | 0·42 | 0·32 |

*Breast cancer mortality rates.*—For 18 countries (Table I, populations C) the correlation with blood group A was negative (—0·18) for the early breast cancer rate and positive (+0·15) for the late rate. The conflict of these results with those obtained for registration rates was thought likely to be a consequence of the different selection of countries which, as can be seen from Fig. 1, could affect the estimated correlation profoundly. The additional evidence presented in the discussion supported the positive correlation with blood group A obtained for registration rates.

*Regression models for breast cancer*

From the foregoing, breast cancer appeared to be associated with three variables, diet, parity and blood group A. The multiple linear regression of the late breast cancer mortality rate for 22 countries on sugar and fat consumptions accounted for 74 % of the total variation (Table V) while, for the early rate, only 46 % was explained (Table V).

TABLE V.—*Regression Model " Breast Cancer = Sugar + Fat + x "*

| Breast cancer | | | | Regression coefficients | | | |
|---|---|---|---|---|---|---|---|
| Data | Rate | n | $R^2$ | Sugar | Fat | x | Constant |
| | | | | | | No x | |
| Mortality | Early . | 22 . | 46 . | 0·25* | 0·31 | — | 7·1 |
| Mortality | Late . | 22 . | 74 . | 1·29* | 1·40* | — | 7·8 |
| Mortality | Early . | 9 . | 71 . | 0·34 | 0·35 | — | 4·1 |
| Mortality | Late . | 9 . | 90 . | 1·78* | 0·86 | — | 0·29 |
| | | | | | | $x = P_0$ | |
| Mortality | Early . | 9 . | 77 . | 0·51* | 0·48 | −83 | 9·1 |
| Mortality | Late . | 9 . | 92 . | 1·45* | 0·61 | 158 | −9·3 |
| | | | | | | No x | |
| Registration | Early . | 13 . | 56 . | 1·42 | 1·38 | — | 22·9 |
| Registration | Late . | 13 . | 78 . | 2·20* | 2·83 | — | 5·7 |
| | | | | | | x = A% | |
| Registration | Early . | 13 . | 64 . | 0·80 | 0·42 | 1·33 | −27 |
| Registration | Late . | 13 . | 81 . | 2·74* | 1·46 | 1·91 | −66 |

* $P < 0\cdot05$.
Units: Rates ($10^{-3}$ females)
Sugar and fat intakes (kg. per year)
$P_0$ (proportion of nulliparous women)

For the 9 countries (Table I, populations B) for which parity estimates were available the regression of the late rate on sugar and fat intakes accounted for 90 % of the variation (Table V) and inclusion in the regression equation of a factor for nulliparity ($P_0$) did not increase $R^2$ substantially (Table V). The contribution of the nulliparity term to the late breast cancer rate was, on average for the 9 countries, one-quarter of that arising from the terms for sugar and fat intakes. The partial regression coefficient for the early rate on $P_0$ was not significant (Table V); it was of interest that the coefficient was negative.

Estimates of the percentage of the population with blood group A were incorporated into the regression equation, in addition to sugar and fat, for the breast cancer registration rates of 13 populations. Inclusion of the term for blood group A increased $R^2$ by 8 % for the early rate and only 3 % for the late rate. From consideration of the partial regression coefficients the average contribution for the 13 countries of blood group A relative to that for sugar and fat together, was $2\frac{1}{2}$ times for the early rate but only one-third for the late rate (Table V).

No attempt was made to examine the effects of parity and blood group A in the same regression equation. For such an analysis only data for 8 countries were available; these were countries listed in Table I (populations C) with the U.S. excepted because no blood group A estimates were available for the whole country. For these 8 populations the breast cancer mortality rates were negatively correlated with blood group A. Because of additional evidence presented in the discussion this correlation was regarded as spurious.

### DISCUSSION

While the early and late breast cancer rates were correlated with one another, and both correlated with sugar and fat intakes, three differences could be recognized. First, the regression on sugar and fat accounted for almost twice the variation in the late rate (74 %) than for the early rate (46 %). Second, as $P_0$, the proportion of nulliparous women in the population, increased, the late breast cancer rate increased but the early rate decreased. Third, for the regression of breast cancer on sugar, fat and blood group A, the contribution of blood group A relative to that for sugar and fat, was about $2\frac{1}{2}$ for the early rate but only one-third for the late rate. These results suggested that the late breast cancer rate was influenced by the environmental factors of diet and factors associated with childbirth, while for the early rate a constitutional factor, blood group A, appeared to be important.

de Waard *et al.* (1960) distinguished early and late breast cancers on physiological grounds. If the foregoing evidence for epidemiological differences were accepted, a consequence would be that failure to take age into account could lead to conflicting results in any study of characteristics of breast cancer. For example, 10 % of all breast cancer deaths in England and Wales occur before the age of 45 years. Assuming that they have the characteristics of the early type then, from de Waard's studies, an additional 10 % could be reasonably supposed to occur symmetrically beyond 45 years of age. The remaining 80 % could then tentatively be ascribed to the late type of breast cancer. Forty-five per cent of all breast cancer deaths in England and Wales occur later than 65 years and if these were regarded as being of the late type, the following picture would emerge:

*Breast Cancer* (*% of all deaths*)

| Age (years) | Early type % | Late type % |
|---|---|---|
| $<45$ | 10 | — |
| 45–64 | 10 | 35 |
| $65<$ | — | 45 |

It is likely that genetic factors are important amongst patients with breast cancer who have a mother or close relative with a history of the disease. In three large series of such cases the age of onset of the disease in the daughter was about a decade earlier than would be expected for all breast cancer patients. For 510 cases (Penrose, Mackenzie and Karn, 1948) the average age at onset was 52 years while the average age at death from breast cancer in the general population was 63 years. Even allowing for the interval between onset and death, this group of breast cancers would be classed as early. For a similar group of 280 breast cancer series (a personal series of Haagensen) Papadrianos, Haagensen and Cooley (1967) found a mean age at onset of 48 years. For comparison the mean age of all breast cancer patients registered in New York during 1960–61 was 60 years (Doll *et al.*, 1966). Jacobsen (1946) found that ages of 154 probands with a hereditary predisposition for breast cancer were significantly younger than for 46 controls. Li and Fraumeni (1969) described 4 families with cancers in children, their parents and near relatives; 3 out of 4 of the mothers had developed breast cancer at an early age. Berg, Hutter and Foote (1968) reported an excess of breast cancer amongst women with a history of salivary gland cancer. For these breast cancers, which could reasonably be supposed to have a contributory genetic factor, the average age at onset was 48 years, about a decade earlier than for breast cancers in the general population.

There would be of course very many possible inherited traits which could enhance development of breast cancer and blood group A would be only one of the possible factors. Three pieces of evidence supported the view that the early rate was positively associated with blood group A. First, registration rates for 13 areas were positively correlated with blood group A. Second, in the multiple regression of the early rate on sugar, fat and blood group A, the partial regression coefficient for blood group A, while not significant, contributed $2\frac{1}{2}$ times more than terms for sugar and fat together; for the late rate the relative contribution of blood group A was only one-third. Additional, if indirect, evidence for an association between blood group A and breast cancer was the reported (Berg *et al.*, 1968) excess of breast cancer amongst women with a history of salivary gland cancer. Salivary gland tumours are themselves associated with blood group A (Cameron, 1958; Osborn and De George, 1961). Hartmann and Stavem (1964) reported a 5% excess of blood group A amongst 1600 patients with breast cancer. This excess was not statistically significant but might be so if the excess of blood group A tended to occur in younger patients.

The association of breast cancer mortality rates with blood group A differed from that for breast cancer registration rates. This conflicting result was thought to be a consequence of the different selection of countries, and the narrower ranges of blood group A values for which breast cancer mortality data were available.

While the early breast cancer rate appeared to have an associated genetic component, environmental factors of parity and diet appeared to exert a stronger effect on the development of breast cancers later in life. The positive association of late breast cancer with nulliparity was in agreement with the findings of Lilienfeld (1963) and Cole and MacMahon (1969). The measure of nulliparity used in this study was an average value for all age groups, and it appeared to make a minor contribution to late breast cancer, relative to that of sugar and fat. Lowe and MacMahon (1970) have demonstrated that the age of the first pregnancy has a marked influence on the effect of child-bearing on the risk of developing breast cancer. A final assessment of the relative contributions of diet and parity to breast cancer would have to take account of the age distribution of first pregnancies.

Previous workers have reported a positive association of total breast cancer with fat intake (Lea, 1966; Carroll, Gammel and Plunkett, 1968; Wynder, 1968). The present study suggested that the influence of fat intake was more important for breast cancer late in life. The positive association with sugar intake, reported here, might be related to Lea's finding (Lea, 1966) that breast cancer mortality was associated with diabetes. Association of breast cancer with a disturbed glucose tolerance has been demonstrated also by de Waard *et al.* (1960). de Waard (1969) has proposed a mechanism whereby overfeeding increasing adrenocortical activity which in turn enhanced the late breast cancer rate.

The author wishes to gratefully acknowledge the painstaking assistance of Miss Alice Duncan.

### REFERENCES

Berg, J. W., Hutter, R. V. P. and Foote, F. W., Jr.—(1968) *J. Am. med. Ass.*, **204**, 113.

Cameron, J. M.—(1958) *Lancet*, i, 239.

Carroll, K. K., Gammel, E. B. and Plunkett, E. R.—(1968) *Can. med. Ass. J.*, **98**, 590.

Cole, P. and MacMahon, B.—(1969) *Lancet*, i, 604.

Doll, R., Payne, P. and Waterhouse, J.—(1966) 'Cancer Incidence in Five Continents'. U.I.C.C. (Springer-Verlag).

Hartmann, O. and Stavem, P.—(1964) *Lancet*, i, 1305.

Jacobsen, O.—(1946) 'Heredity in Breast Cancer'. London (H. K. Lewis, Ltd.).

Lea, A. J.—(1966) *Lancet*, ii, 332.

Li, F. P. and Fraumeni, J. F.—(1969) *Ann. intern. Med.*, **71**, 747.

Lilienfeld, A. M.—(1963) *Cancer Res.*, **23**, 1503.

Lowe, C. R. and MacMahon, B.—(1970) *Lancet*, i, 153.

Mourant, A. E., Kopec, A. C. and Domaniewska-Sobczak, K.—(1958) 'The ABO Blood Groups'. Oxford (Blackwell).

Osborne, R. H. and De George, F. V.—(1961) *Proc. 2nd. Int. Conf. Human Genetics* (Johns Hopkins).

Papadrianos, E., Haagensen, C. D. and Cooley, E.—(1967) *Ann. Surg.*, **165**, 10.

Penrose, L. S., Mackenzie, H. J. and Karn, M. N.—(1948) *Br. J. Cancer*, **2**, 168.

Segi, M. and Kurihara, M.—(1966) 'Cancer Mortality for Selected Sites in 24 Countries', No. 4. Japan (Sendai).

Segi, M., Kurihara, M. and Matsuyama, T.—(1969) 'Cancer Mortality for Selected Sites in 24 Countries', No. 5. Japan (Sendai).

United Nations—(1950–65) *Statistical Yearbook*. United Nations.

UNITED NATIONS—(1959) *Demographic Yearbook.* United Nations.
DE WAARD, F., DE LAIVE, J. W. J. AND BAANDERS-VAN HALEWIJN, E. A.—(1960) *Br. J. Cancer*, **14**, 437.
DE WAARD, F.—(1969) *Int. J. Cancer*, **4**, 577.
WYNDER, E. L.—(1968) 'Proceedings of the first Tenovus Symposium', 1967, edited by A. P. M. Forrest and P. B. Kunkler. Cardiff (E. & S. Livingstone).
WYNDER, E. L., BROSS, I. J. AND HIRAYAMA, T. A.—(1960) *Cancer, N.Y.*, **13**, 559.